# Response to Letter to the Editor: “Erector spinae plane block versus caudal block in children: similar analgesia, different stories beneath the surface”

**DOI:** 10.1016/j.bjane.2026.844784

**Published:** 2026-07-11

**Authors:** Barbara Bombassaro Masiero, Rafael Arsky Lombardi

**Affiliations:** a Pontifícia Universidade Católica do Rio Grande do Sul, Porto Alegre, RS, Brazil; b University of Nebraska, Lincoln, Kansas, United States of America

Dear Editor,

We thank Dr. Mistry and Dr. Nair for their thoughtful Letter to the Editor^1^ regarding our systematic review and meta-analysis comparing erector spinae plane block and caudal epidural block in pediatric surgery.^2^ Their comments provide an important opportunity to clarify the interpretation of our findings, particularly regarding the certainty of evidence, heterogeneity, and anatomical classification of ESPB techniques.

Regarding the reported inconsistency in the certainty of evidence,^1^ we acknowledge the discrepancy between the abstract and the GRADE assessment in the main text. As noted, the primary outcome (time to first rescue analgesia) was supported by low-certainty evidence due to heterogeneity and imprecision, while higher certainty applied only to selected secondary outcomes. We agree that this distinction is important, and conclusions regarding equivalence should be interpreted in this context. This reflects an inconsistency in reporting rather than differences in the underlying analyses.

We also appreciate the comments regarding the significant heterogeneity observed in the primary outcome (I² = 99%).^1^ This limitation was acknowledged and discussed in the manuscript and reflects the clinical diversity of the included studies, including differences in patient populations, surgical procedures, block levels, and anesthetic techniques.^2^ Subgroup analyses were not feasible due to inconsistent reporting of key variables and the limited number of studies per comparison.^2^ Sensitivity analyses showed that no single study disproportionately influenced the overall results.^2^

Regarding the classification of ESPB, we acknowledge that sacral, lumbar, and thoracic approaches may differ in anatomical spread and clinical application.^2^ These differences were considered part of the clinical heterogeneity. ESPB was treated as a single intervention based on its shared underlying principle as a fascial plane block and a similar technical approach.^2^ We agree that further stratification by block level may provide additional insights when sufficient data are available.

Finally, we agree that broader clinical and patient-centered outcomes would enhance applicability. However, these variables, as well as factors influencing POUR such as neuraxial opioid use and surgical type, were inconsistently reported across the included trials and not available in sufficient detail for meaningful analysis.^3^, ^4^, ^5^, ^6^, ^7^, ^8^, ^9^, ^10^, ^11^ These limitations reflect gaps in the available literature and highlight the need for more standardized reporting in future studies.

We appreciate the opportunity to clarify these points.

## Use of AI and AI-assisted technologies

The authors declare that no artificial intelligence or AI-assisted technologies were used in the preparation of this manuscript.

## Data availability statement

No new data were created or analyzed in this article. Data sharing is not applicable to this article.

## Author’s contributions

Barbara Bombassaro Masiero: Conceptualization; data curation; methodology; writing-original draft; project administration. Rafael Arsky Lombardi: Conceptualization; data curation; methodology; writing; project supervision; validation.

## Funding

This research received no grant from any funding agency.

## Conflicts of interest

All authors report no relationships that could be considered conflicts of interest. All authors take responsibility for all aspects of the reliability and freedom from bias of the data presented and their discussed interpretation.
